# Supplementary material for: Botulinum toxin injection without electromyographic guidance in consecutive esotropia
Source: PLoS One. 2020 Nov 12;15(11):e0241588. doi: 10.1371/journal.pone.0241588 (PMC7660504; doi:10.1371/journal.pone.0241588)
Supplement: Attachment — Submitted filename: Response_to_Reviewers 2.docx [file pone.0241588.s002.docx]

September 4, 2020

I-Jong Wang

Academic Editor

*PLOS ONE*

Ref: PONE-D-20-07281R1

­­­­­­­­­­­­­­­­­­­Botulinum toxin injection without electromyographic guidance in consecutive esotropia

Dear Dr. I-Jong Wang:

I thank the editors and reviewers for taking their time to review our manuscript entitled "­­­­­­­­­­­­­­­­­­­Botulinum toxin injection without electromyographic guidance in consecutive esotropia”. A response to the reviewers’ comments/requests in accordance with the Editorial Board Member’s recommendation is included below. Each of the coauthors has seen and agreed with the changes made to this revision. I hope the revised manuscript will better meet the publication requirement of *PLOS ONE*.

Sincerely,

Jeong-Min Hwang, M.D., PhD

Professor

Department of Ophthalmology,

Seoul National University College of Medicine

Seoul National University Bundang Hospital

Reviewer #1: Dear Authors,
Thanks for your responses and explanations in detail.
Botulinum injection without EMG guide indeed needs greater care of the localization of the injection site very precisely. Thanks for sharing this method and your outcomes in treating consecutive esotropia with us. There are several points need to be explained more clearly.

1. As the authors mentioned, the dosage of the Botulinum injection differed throughout the whole study. Therefore, it is difficult to compare them all on the same basis. Please persuade us that they are comparable in the discussion.

Response

Thank you very much for your advice. We added the following sentences in the discussion (line 294 - 298) as follows:

“Regarding the unequal dosage of botulinum toxin throughout the study, we incorporated the mean dosage of botulinum toxin per 10 PD of esodeviation for analysis. However, there was no significant difference in the mean dosage of botulinum toxin between the success and failure groups.“

2. How about classifying them into subgroups of different doses of Botulinum to see if there are any differences in the success/failure rate and to clarify the optimal dosage.

Response

We tried several analyses concerning the dosage of botulinum toxin and incorporated the mean dosage of botulinum toxin per 10 PD of esodeviation for analysis, however there was no difference between the success and failure groups. Also, the number of patients was too small to subgroup patients into different dosages.

3. The differences of the dosages of Botulinum and treatment outcomes between previous EMG-guided injection studies and the authors' study should be mentioned.

Response

We added the following contents in the discussion (line 283 - 287) as follows:

“The average dose of botulinum toxin injection in our study (3 to 6 IU per muscle) was slightly larger than that in previous studies using electromyographic guidance in which the mean dosage ranged between 1.25 to 5.0 IU per muscle, whereas the overall success rate in our study was comparable to the former studies ranging from 67 to 86%. [8,10]

We deeply appreciate your precious time and effort for the manuscript.

Reviewer #2: The authors had adequately addressed all my comments and revised their manuscript. Only two trivial questions remained.

1.In table 2, the variables "Immediate success at 2 weeks after injection " is duplicated.

Response

Thank you very much for your careful review. We deleted the last line in Table 2.

2.The revised manuscripts showed that initial postoperative esodeviation of less than 18 PD at one month after exotropia remained a significant predictive factor of success. I am interested in how the authors determine this cut off value of PD. (for example, via ROC curve analysis)

Response

We evaluated the significance of different cutoff values of initial postoperative esodeviation between 15~20 PD through univariate analysis.

Reviewer #4: The author has answered most of the questions proposed, however, in table 2, the last line duplicates the 3rd line and should be revised.

Response

We deleted the last line in Table 2. Thank you for the careful review.

Reviewer #5: It is great that those 3 patients with 6-8 PD of esotropia before injection had improved after Botulinum toxin injection. However, it is controversial to include these 3 cases who meet the definition of the success group at the beginning.

Response

As per the reviewer’s suggestion, we excluded the 3 patients with a small angle of esodeviation less than 10 PD and performed the statistical analyses again. We are sorry for the delay because of the statistical analyses. We changed all the results accordingly.

We deeply appreciate your careful review.

Reviewer #6: Thank you for the revision.
Please clarify the Esodeviation at near before injection in the abstract.

Response

Thank you for your comment. We added the “esodeviation at near” in the abstract.
